# Altered Hepatic and Cerebral Lipid Mediator Pathways in Alzheimer’s Disease

**DOI:** 10.1002/alz.086467

**Published:** 2025-01-03

**Authors:** Sitong Zhou, Kamil Borkowski, Nuanyi Liang, Thomas G. Beach, Geidy E Serrano, Kyoungmi Kim, Bruce D. Hammock, John W. Newman, Lee‐Way Jin, Izumi Maezawa

**Affiliations:** ^1^ University of California Davis Medical Center, Sacramento, CA USA; ^2^ University of California, Davis, Davis, CA USA; ^3^ Civin Laboratory for Neuropathology, Banner Sun Health Research Institute, 10515 W Santa Fe Drive, Sun City, AZ 85351, Sun City, AZ USA; ^4^ United States Department of Agriculture, Agriculture Research Service, Davis, CA USA; ^5^ University of California Davis Medical Center, Davis, CA USA

## Abstract

**Background:**

Inflammation is crucial in Alzheimer’s Disease (AD), where oxidized lipid derivatives of polyunsaturated fatty acids (PUFAs), i.e., oxylipins, are potent modulators. Soluble epoxide hydrolase (sEH), known for converting pro‐resolving epoxy‐fatty acids to pro‐inflammatory dihydroxy‐fatty acids (diols), is upregulated in the AD brain. Recent studies identify AD as a systemic disorder, but peripheral organs are understudied. Given the liver’s pivotal role in metabolizing lipids and circulating lipid mediators’ production, we tested the hypothesis that the inflammatory lipid mediator networks altered in the brain are also perturbed in the liver.

**Method:**

AD diagnosis was based on the NIA‐AA guidelines. We analyzed lipid mediators in paired postmortem brain and liver tissues from 16 cognitive normal and 63 AD participants with short postmortem intervals (≤3 hours). Both alkali‐releasable and free oxylipins were measured using LC‐MS/MS‐based methods. Aberrant metabolites associated with AD were identified using analysis of covariance, adjusting for sex, age, BMI, and postmortem interval. Sex differences were explored.

**Result:**

In both the brain and liver of AD participants, we found altered levels of alkali‐releasable ethanolamides, recognized for their neuroprotective and anti‐inflammatory properties. The most significant changes were observed in sEH activities within the AD liver, where pro‐inflammatory diols decreased in alkali‐releasable fractions. This reversely confirms the published results of increased free diols in AD plasma, as the esterified oxylipin pool serves as a reservoir for free oxylipins. These findings suggest that circulating metabolites of sEH and ethanolamides may predict AD pathogenesis. We also found that the decreased level of pro‐inflammatory 20‐HETE in the AD liver, which did not align with our expectations. Moreover, observations hinted at male proneness to inflammation.

**Conclusion:**

Our data show significant alterations in lipid mediators in both the AD brain and liver, with liver exhibiting the most pronounced changes. This supports AD being a systemic disorder with the potential for liver to regulate brain function via lipid mediators and emphasizes the complexity of lipid metabolism in AD. Sex distinctions underscore the importance of considering it as a variable in future research. Additionally, it highlights the need to explore the biological implications of alkali‐releasable mediators.

